# Evaluating the “return on patient engagement initiatives” in medicines research and development: A literature review

**DOI:** 10.1111/hex.12951

**Published:** 2019-09-06

**Authors:** Lidewij Eva Vat, Teresa Finlay, Tjerk Jan Schuitmaker‐Warnaar, Nick Fahy, Paul Robinson, Mathieu Boudes, Ana Diaz, Elisa Ferrer, Virginie Hivert, Gabor Purman, Marie‐Laure Kürzinger, Robert A. Kroes, Claudia Hey, Jacqueline E.W. Broerse

**Affiliations:** ^1^ Athena Institute Vrije Universiteit Amsterdam Amsterdam The Netherlands; ^2^ Nuffield Department of Primary Care Health Sciences University of Oxford Oxford UK; ^3^ MSD (Merck Sharp & Dohme) London UK; ^4^ European Patients' Forum (EPF) Brussels Belgium; ^5^ Alzheimer Europe Luxembourg Luxembourg; ^6^ EURORDIS – Rare Diseases Europe Paris France; ^7^ Nexgen Healthcare Communications London UK; ^8^ Sanofi Chilly-Mazarin France; ^9^ Lilly Nederland BV Utrecht The Netherlands; ^10^ Merck Healthcare KGaA Darmstadt Germany

**Keywords:** evaluation, framework, impact, literature review, medicines development, metrics, patient and public involvement, patient engagement, patient participation, research

## Abstract

**Background:**

Showing how engagement adds value for all stakeholders can be an effective motivator for broader implementation of patient engagement. However, it is unclear what methods can best be used to evaluate patient engagement. This paper is focused on ways to evaluate patient engagement at three decision‐making points in the medicines research and development process: research priority setting, clinical trial design and early dialogues with regulators and health technology assessment bodies.

**Objective:**

Our aim was to review the literature on monitoring and evaluation of patient engagement, with a focus on indicators and methods.

**Search strategy and inclusion criteria:**

We undertook a scoping literature review using a systematic search, including academic and grey literature with a focus on evaluation approaches or outcomes associated with patient engagement. No date limits were applied other than a cut‐off of publications after July 2018.

**Data extraction and synthesis:**

Data were extracted from 91 publications, coded and thematically analysed.

**Main results:**

A total of 18 benefits and 5 costs of patient engagement were identified, mapped with 28 possible indicators for their evaluation. Several quantitative and qualitative methods were found for the evaluation of benefits and costs of patient engagement.

**Discussion and conclusions:**

Currently available indicators and methods are of some use in measuring impact but are not sufficient to understand the pathway to impact, nor whether interaction between researchers and patients leads to change. We suggest that the impacts of patient engagement can best be determined not by applying single indicators, but a coherent set of measures.

## 1 INTRODUCTION

There is increasing consensus among stakeholders that patient engagement in research and development (R&D) of medicines provides benefits for patients, researchers, industry, regulatory bodies, payers and policy makers.^1^, ^2^, ^3^ The case for patient engagement is often presented in ethical and political terms referring to fairness, transparency and accountability.^4^, ^5^ Methodological arguments consider the experiential knowledge of patients—acquired by their personal experience of a condition—as valuable to improving the quality and relevance of the research.^6^, ^7^, ^8^ The inclusion of patients in decision making about the development of new innovative medicines is a substantial change, requiring time and (financial) commitments from researchers, industry and patients.^2^, ^4^ Despite efforts to promote and support patient engagement in research, the prevalence of patient engagement in medicines research and development remains low.^9^, ^10^ Patient engagement has not been fully embedded in the health research system, partly because it is not yet clear to all involved what the added value is.^11^ To address this need, an increasing number of studies aim to evaluate the impact of patient engagement, underscoring the growing interest in the “return on engagement,” or why it makes sense for patients, society and industry.^2^, ^12^

The perceived value of patient engagement practices can vary for different stakeholder groups, and the metrics of interest will therefore differ accordingly.^13^ For example, for researchers and industry partners it might be about evidence that patient engagement improves the quality and efficiency of research and the uptake of findings, whilst for patients it might be more about influencing the R&D agenda to develop medicines for unmet needs. Some argue that evidence is needed to justify the ‘business case’ for engagement. This could also help to establish a financial model to support engagement.^2^, ^14^, ^15^ Evaluation could also define the genuine value of patients’ contributions, contributing to valued rather than tokenistic inclusion for appearances’ sake.^16^ There is also some resistance; people are concerned about assessing impact too simplistically. Some question whether it is fair to evaluate the value of patient input in isolation, and not that of others such as key scientific leaders,^12^ not least because it may be the synergy of working in partnership that produces benefit.^17^ As mentioned by Staniszewska, it is important to recognize that “any form of measurement sits within a political or ideological context that cannot be ignored.”^13^ Nonetheless, there is a desire to assess the impact of patient engagement, to demonstrate better decision making, avoidance of previous errors and a contribution to continuous efficiency and quality improvement.^15^, ^16^, ^18^

Despite this perceived importance of assessing the return on patient engagement, little is known about “what” to evaluate, and even less about “how.”^19^, ^20^, ^21^ A number of researchers have tried to assess how patient engagement makes a difference.^3^, ^5^, ^8^, ^12^, ^22^, ^23^, ^24^, ^25^, ^26^, ^27^ Although there is no standardized way to assess the impact of patient engagement, very similar benefits, costs and challenges are reported in literature reviews.^4^, ^17^, ^19^, ^20^, ^28^, ^29^, ^30^, ^31^, ^32^ The current assessment of patient engagement is considered weak, partly because much of the evidence is mainly anecdotal^17^ and because methods used have not captured the complexity, context or mechanisms of change.^17^, ^33^ Previous studies have identified a number of gaps in the literature and identified challenges such as the delayed nature of impact, inconsistent terminology, absence of accepted criteria for judging the success or quality of research, no agreed evaluation methods or framework and few reliable measurement tools. The absence of a control group—identical research carried out without patient engagement—is problematic too, particularly in an area of science where direct comparison to an existing standard is routinely demanded.^8^, ^16^, ^34^, ^35^ It is argued that to build an evidence base, some level of consensus on measurable impacts is needed, whilst others state that the outcomes of engagement cannot easily be quantified.^13^, ^30^, ^36^ In sum, it remains unclear what methods can be best used to evaluate patient engagement.

To address the need for means of determining the “return on engagement,” the aim of this paper was to scope, review and summarize the literature on monitoring and evaluation of patient engagement. Many publications present useful guidance for conducting patient engagement and assessing the quality.^37^, ^38^, ^39^ Evaluation studies focus mainly on qualitative methods and only occasionally link to specific outcomes.^12^, ^33^, ^40^, ^41^ Therefore, this paper is focused on ways to evaluate patient engagement with both qualitative and explicitly quantitative methods.

This work is part of the PARADIGM project, a public‐private partnership that is developing ways to ensure that patients are always meaningfully involved in the development of medicines. The impact of patient engagement may differ at different points in the development of a medicine. Accordingly, PARADIGM focuses on three decision‐making points during R&D at which point integration of the patient perspective is considered likely to be valuable, specifically as part of research priority setting, design of clinical trials and at early dialogues with regulators and health technology assessment bodies. Each of these represents a point at which engagement can influence effective planning and implementation, and demonstrate impact on the final product.

## 2 METHODS

We undertook a scoping review of published academic and grey literature as recommended by Arksey and O’Malley, also drawing on Mays et al and Peters et al^42^, ^43^, ^44^ Scoping reviews are similar to systematic reviews in that they follow a structured search process; however, they are performed for different reasons.^45^ Our aim was not to answer a precise question addressing the effectiveness of a certain practice, as in a meta‐analysis, but to provide an overview of the breadth of the available literature about evaluating patient engagement.

Whilst the review is concerned with patient engagement at the three key decision‐making points, we used broader search limits to ensure capture of related publications in other areas of health research. One of the challenges was the variety of terminology. For example, the words “measure,” “metric” and “indicator” are often used interchangeably and their definitions may vary, if they are stated at all. Furthermore, the terms used for “patient engagement” differ globally. In this paper, we use the term patient engagement; in our search, we included terms such as public involvement, patient participation, community engagement and user involvement. In Table 1, we provide definitions of terms developed by the authors and as we used them in this review.

**Table 1:** Definitions

Concept	Description
Patient engagement	The effective and active collaboration of patients, patient advocates, patient representatives and/or carers in the processes and decisions within the medicines lifecycle, along with all other relevant stakeholders when appropriate^1^
Patient partner	A patient, patient advocate, patient representative and/or carer who contributes to any level of patient engagement activities; this can also be substituted for other terms such as patient contributor^82^
Research participant	A person who participates in human subject research, also called a subject, study participant or volunteer of an experiment or trial
Society	Includes all members of the public and patients who use health‐care services
Research priority setting	Any process aimed at constructing priorities or agendas for health research and medicines development, to raise awareness and change the way research funding is allocated
Design of clinical trials	Any process aimed at the development or design of clinical trials for medicines development at any stage of that process. One example is changes made to inclusion and exclusion criteria for trial participants
Early dialogues with regulators and Health Technology Assessment (HTA) bodies	Any process in which medical technology developers communicate with regulatory bodies and/or HTA bodies prior to health technology assessment. Early dialogue can happen only with regulators (eg scientific advice), jointly with regulators and HTA bodies (to discuss data requirements to support decision making on marketing authorization and reimbursement simultaneously) or only with HTA bodies (eg EUnetHTA multi‐HTA dialogues)
Benefit	An advantage of engagement for research and development and stakeholders involved
Costs and challenges	The expenditure and/or effort of engagement for research and development and the stakeholders involved
Outcomes	Decisions made and things produced as a direct result of patient engagement practices. One example is changes made in the design of a clinical trial resulting in a more relevant and appropriate research protocol. Outcomes may lead to impact on research and development
Impacts	Broader effect of outcomes, both positive and negative, of patient engagement. Impact may be direct or indirect, intended or unintended. For example, this may include study quality benefits such as improved recruitment and retention of study participants
Value	The benefits of patient engagement (in relation to the direct and indirect costs) for individuals and organizations involved
Monitoring	The formative evaluation of patient engagement practices in order to strengthen them
Evaluation	The ‘systematic acquisition and assessment of information to provide useful feedback about …’ patient engagement practices.^83^ Summative evaluation examines the effects of patient engagement practices on various measures including outcomes, impact and cost‐benefit
Criteria	Dimensions or parameters used for evaluation. These need to be translated into measurable entities called ‘indicators’ and indicators are measured with ‘metrics’
Indicator	Qualitative or quantitative measure that provides a means of expressing achievement of a goal or ascertaining the consequences of a specific change. Quantitative indicators are reported as numbers, such as rates of change and ratios. Qualitative indicators are reported as words, in statements, paragraphs and reports^84^
Metrics	Observations based on standardized data sources or agreed techniques for gathering information. Metrics could consist of an agreed set of quantitative and/or qualitative indicators to measure evaluation criteria, with a set of agreed methods/tools to collect this information
Methods	Ways to collect information for monitoring and evaluating the outcomes and impact of patient engagement practices, for example quantitative, qualitative or mixed methods
Tools	Instruments to collect information about patient engagement practices. For example, interview guides, questionnaires, log sheets and observation forms are all tools

### 2.1 Search methods

Prior to the database search, we did a search to identify a tentative sample set of relevant studies for a snowballing exercise. Using broad key words, we searched Google Scholar for published articles and Google for grey literature. We also searched the Patient‐Centered Outcomes Research Institute (PCORI) database^46^ and the INVOLVE evidence library.^47^ A snowballing exercise using references and citations from these articles provided a starting set of publications that informed the protocol for the main review. This is recommended for the clarification of concepts and search terms when interrogating large, diverse fields of literature.^48^

Accordingly, with the assistance of a specialist librarian, we searched CINAHL, Embase, Medline, PsychINFO and PubMed databases for peer‐reviewed published literature. The following key words were used “patient engagement” combined with « AND» “research” « AND» “outcomes,” including a variation of terms combined with « OR». An overview of all search terms can be found in Table 2.

**Table 2:** Search terms

Patient engagement (title only)	Research (title only)	Outcomes (title/abstract only)
Patient participation [MeSH]	Comparative effectiveness research [MeSH]	Outcome(s) Impact
Patient engagement	Research	Measurement(s)
Public engagement	Clinical trial	Metrics
Client engagement	Study design	Framework(s)
Community engagement	Trial design	Assessment
Public participation	Research design	Criteria
Patient participation	Health technology assessment	Indicator(s)
Public involvement	Agenda setting	
User involvement		
Client involvement		
Consumer involvement		

Grey literature was searched using the same terms; items recommended by consortium partners and external stakeholders were added manually, and reference lists of items included were searched for additional publications. All searches were conducted between 1 May 2018 and 31 July 2018. The search was limited to publications in English. We excluded articles that did not provide information on possible evaluation approaches or outcomes associated with patient or public engagement. No date limits were applied other than a cut‐off of publications after 31 July 2018. Following completion of the search, duplicated items were removed.

### 2.2 Study selection and data extraction

**Figure 1:**
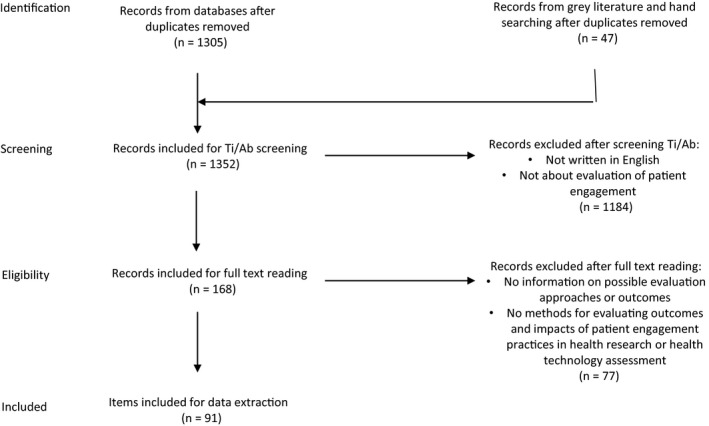
Article selection PRISMA flow diagram

Two researchers (TF, LV) independently screened all items’ title and abstract. To ensure inter‐rater reliability, items were marked for inclusion or exclusion with each researcher's initials, discrepancies were discussed and consensus reached. Both researchers read all the selected items in full and followed up references for final inclusion. At this stage, further exclusions were made of items that did not include methods for evaluating outcomes and/or impact of patient engagement practices in health research or health technology assessment—discrepancies were discussed, and consensus agreed for final inclusion in the data extraction and analysis. Figure 1 demonstrates the number of articles identified, screened, selected and reviewed.

TF and LV developed a data extraction sheet to record relevant information from each item, including the publication year and focus, country of origin, methodology, patients involved as partners, use of a framework or model, definitions included, outcome and/or impact on research, benefits and costs per stakeholder group, measurement or evaluation methods suggested or applied. When available, context and process criteria were also included. Both researchers extracted data independently from 30% of the scientific articles and then compared their findings to agree the approach to data extraction. Thereafter, all the peer‐reviewed published papers’ data were extracted by LV and the grey literature by TF and LV.

### 2.3 Analysis

Data were thematically analysed following Braun and Clarke's approach.^49^ To achieve the summary, we coded data using the review question, aim and objective as included on the data extraction sheet. Codes used were benefits (B), costs and challenges (C), outcome or impact on research (O or I) and types of benefits or costs and challenges. Codes were then clustered into themes, which were agreed by TF and LV; themes were identified deductively and include benefits, costs and challenges for each stakeholder group, benefits, costs and challenges for research per decision‐making point, indicators, methods or tools. LV and NG clustered the indicators, methods and tools into qualitative and quantitative types. Benefits and costs were mapped to suggested or applied indicators and tools or methods. The decision‐making point focus of articles was interpreted by the researchers if not defined in the article. Benefits and costs that could not easily be linked to one particular decision‐making point were analysed separately. LV and TF agreed on the data analysis strategy, and sections of the analysis were cross‐checked by comparing interpretations of results; inconsistencies were discussed and agreed.

### 2.4 Consultation and validation

The preliminary results of the review were presented and discussed during a PARADIGM meeting held in London, 18 July 2018. This session provided valuable input on how best to present and categorize the results. Participants in the meeting included representatives from patient organizations, pharmaceutical companies and academia with an interest and considerable expertise in patient engagement. Based on the discussions during the meeting, it was agreed to structure the results per key decision‐making point and per stakeholder, including benefits and costs. These members of the PARADIGM consortium were involved in writing this article; their interpretation of results informed the discussion and conclusion. Furthermore, their contributions to the entire research process informed the direction of research, the terminology and definitions used in this article.

## 3 RESULTS

A total of 91 documents met the eligibility criteria (academic literature n = 77 and grey literature n = 14). Included documents were published between 2000 and 2018 and focused mainly on the health research field. We found limited documents in the field of regulation and health technology assessment. Most documents were published in the United Kingdom in an academic setting. We found largely qualitative studies and literature reviews. Sixteen studies reported that patients were involved in the study as partners. Table 3 provides an overview of the characteristics of included documents.

**Table 3:** Overview of characteristics of included documents

Characteristic	Output
Year	Last 8 y (2010‐2018) (n = 69)
	10 y (2000‐2010) (n = 22)
Focus	Clinical trial (n = 24)
	Health research (n = 47)
	Regulation and HTA (n = 8)
	Other (n = 12)
Country of origin	Canada (n = 11)
	United Kingdom (n = 40)
	Canada and the United Kingdom (n = 1)
	United States (n = 27)
	Europe (n = 6)
	Netherlands (n = 4)
	Germany (n = 1)
	Denmark (n = 1)
Setting	Academia (n = 38)
	Health care (n = 19)
	Industry (n = 3)
	Mixed (n = 14)
	Other (n = 17)
Methodology (academic literature only)	Quantitative study (n = 6)
	Qualitative study (n = 23)
	Mixed method study (n = 15)
	Literature review (n = 25)
	Commentary/Editorial/Opinion/other (n = 8)
Patients involved as partners in the study (academic literature only)	Yes (n = 16)
	Unspecified (n = 61)

In this section, we present the findings of our review, first considering the three decision‐making points, which were relevant to our search. Not all reported benefits and costs could easily be linked to one decision‐making point. These referred to overall benefits and costs for stakeholders and general costs or challenges for research and development. Therefore, we report them separately.Additionally, reported benefits and costs were omitted where they related to other phases of the research process (such as interpretation of research findings or dissemination of results).

### 3.1 Benefits, costs and challenges for research and development

A total of 18 benefits and five costs of patient engagement at three R&D decision‐making points were identified. These were grouped into 11 domains and mapped with 28 possible indicators for their evaluation. Tables 4 and 5 provides an overview of indicators per domain. Please refer to Appendices S3–S5 for more detailed indicators, evaluation methods and tools.

**Table 4:** Summary of benefits for research and development mapped with reported indicators for evaluation

**Research priority setting**	
***Usability benefits (1)***	*Examples of indicators related to usability benefits (total: 6)*
More relevant research topics and priorities, based on patients’ needs^3^, ^4^, ^15^, ^17^, ^20^, ^22^, ^23^, ^29^, ^30^, ^50^, ^51^, ^52^, ^53^, ^54^, ^55^	Rating of influence of patients and other stakeholders^61^ Rating of relevance or importance of studies^23^, ^59^ Perceptions or degree of contentment/satisfaction with the topic generation and prioritization process^96^ Similarities and differences in research priorities between stakeholder groups^15^ Types of research gaps reported that were not previously identified^61^ Perceptions on how patients’ experiential knowledge helped shaped the research question^30^
Research questions, hypothesis, interventions and medical technologies become more relevant and usable for patients^24^, ^30^	
***Societal benefits (2)***	*Examples of indicators related to societal benefits (total: 3)*
More appropriate resource allocation, based on patients’ needs^30^	Comparison of academic and lay scores assigned to research proposals^60^ Perceptions of public influence on funding decisions^60^ Indicators of dynamics in the panel discussion^61^
***Funding benefits (3)***	*Examples of indicators related to funding benefits (total: 1)*
Improved fundability and credibility of research proposals^25^, ^29^, ^30^, ^31^, ^32^, ^56^, ^57^, ^58^	Number of studies that had success in gaining research funding^12^
**Design of clinical trials**	
***Ethical benefits (4)***	*Examples of indicators related to ethical benefits (total: 1)*
More appropriate, inclusive and sensitive research design^8^, ^17^, ^29^, ^30^, ^52^, ^55^, ^58^	Number of studies that had success in gaining ethics approval^12^
***Methodological benefits (5)***	*Examples of indicators related to methodological benefits (total: 4)*
More appropriate wording and timing of research instruments and interventions^17^, ^20^, ^22^, ^24^, ^25^, ^27^, ^29^, ^31^, ^55^, ^56^, ^64^, ^65^, ^66^, ^67^, ^68^	Number of changes made to clinical trial communication as a result of study participant feedback^59^
Increased readability and accessibility of research materials^4^, ^20^, ^24^, ^25^, ^29^, ^31^, ^40^, ^55^, ^56^, ^67^, ^68^	Reading level of research documents/instruments^70^ Rating or perceptions of understanding of the consent form^70^
More relevant research outcomes/endpoints^32^, ^41^, ^93^	Number and type of patient‐reported outcomes^61^
***Study quality benefits (6)***	*Examples of indicators related to study quality benefits (total: 7)*
Improved recruitment and retention^23^, ^24^, ^29^, ^40^, ^69^	Recruitment rates^40^, ^69^, ^70^ Number of study participants who dropout for reasons other than adverse reactions^59^
Increased diversity of study participants^66^	Recruitment and retention rates among hard‐to‐reach population, level of diversity^61^
Improved trial experience/satisfaction by study participants^2^, ^80^	Rating or explore feelings of satisfaction among study participants^15^, ^70^ Rating convenience of study visits and procedures by study participants^59^
More adherence to the research protocol^93^	Number of protocol amendments^59^
Faster study completion^2^, ^23^	Number of studies completed within a particular timeframe^3^, ^61^
**Regulatory and HTA processes**	
***Instrumental benefits (7)***	*Examples of indicators related to instrumental benefits (total: 1)*
Higher accuracy in measuring needs and preferences of patients^71^, ^72^	Perceptions on how patient input was used and added value for assessment^75^, ^76^
Better quality of assessment (in terms of relevance and reliability to local context)^71^, ^72^	
***Study uptake benefits (8)***	*Examples of indicators related to study uptake benefits (total: 2)*
Uptake of evidence/approval by regulators and HTA bodies^2^, ^73^	Time to approval/response of regulators^52^ Changes in the proportion of drugs recommended for reimbursement^36^
***Developmental benefits (9)***	*Examples of indicators related to developmental benefits*
Knowledge and public awareness of products^72^	None reported
Democratic accountability and transparency^72^	

**Table 5:** Summary of costs for research and development mapped with reported indicators for evaluation

**Various decision‐making points**	
*Non‐financial costs (10)*	*Examples of indicators related to non‐financial costs (total: 2)*
Biases in recruitment or findings^24^, ^67^	Perceived negative impacts of patient engagement for research and development^24^ Total hours spent on engagement^24^
Scientific and ethical conflict in protocol design^20^	
Power struggles^20^	
Increased time^20^	
***Financial costs (11)***	*Examples of indicators related to financial costs (total: 1)*
Increased costs^20^	Total monetary costs of engagement for research and development^24^

#### 3.1.1 Benefits of patient engagement in research priority setting

Literature suggests that patient engagement in research priority setting has several benefits. We identified four unique benefits and nine possible indicators. We clustered the benefits into three domains: usability benefits, societal benefits and funding benefits. Usability benefits refer to impact on the topic generation and prioritization process, for example more relevant topics and priorities based on patients’ needs^3^, ^4^, ^15^, ^17^, ^20^, ^22^, ^23^, ^29^, ^30^, ^50^, ^51^, ^52^, ^53^, ^54^, ^55^ and the relevance of studies, for example more relevant research questions and medical interventions or technologies.^30^ Societal benefits refer to the way public and private resources are allocated, for example more appropriate resource allocation based on patients’ needs.^30^ Funding benefits refer to new funding and funding opportunities, for example success in gaining funding due to enhanced credibility of research proposals.^25^, ^29^, ^30^, ^31^, ^32^, ^56^, ^57^, ^58^

In the literature, quantitative methods are used to collect information about the perceived importance of studies by patients, the perceived influence of stakeholders in research priority setting,^23^, ^59^ or to compare academic and lay scores assigned to research proposal evaluation.^60^ For example, studies suggest rating the importance or influence of partners in developing the research topics.^23^, ^59^ Qualitative methods are used to explore the relevance of research topics and how patients’ experiential knowledge helped shape the research question.^30^ The Patient‐Centered Outcome Institute (PCORI) uses mixed methods (survey, focus groups, database review) to explore the perceptions incorporated into the topic selection process and the kinds of research gaps documented as important to patients and other stakeholders that were not previously identified.^61^ Quantitative methods could also be used for comparison of academic and lay scores assigned to research proposals.^60^ Qualitative methods are suggested for exploring similarities and differences in research priorities.^15^ For example, Brown et al invited patients with diabetes to focus groups to identify research priorities. Results were analysed using the constant comparative method and compared with current expert‐led research priorities in diabetes.^62^ Additionally, documentary analyses (eg review of minutes, grant applications, reports) may be conducted to compare patient input and responsiveness to patients’ ideas.^54^, ^61^, ^63^

#### 3.1.2 Benefits of patient engagement in the design of clinical trials

We identified ten unique benefits of patient engagement for the design of clinical trials, including 13 possible indicators. We clustered the benefits into three domains: ethical benefits, methodological benefits and study quality benefits. Several studies described ethical benefits such as a more appropriate, inclusive and sensitive research design.^8^, ^17^, ^29^, ^30^, ^52^, ^55^, ^58^ Furthermore, studies described methodological benefits such as more appropriate wording and timing of research instruments and interventions,^17^, ^20^, ^22^, ^24^, ^25^, ^27^, ^29^, ^31^, ^55^, ^56^, ^64^, ^65^, ^66^, ^67^, ^68^ and improved consent forms and accessible recruitment materials.^4^, ^20^, ^24^, ^25^, ^29^, ^31^, ^40^, ^55^, ^56^, ^67^, ^68^ Study quality benefits are also reported, for example improved trial recruitment and retention.^23^, ^24^, ^29^, ^40^, ^69^

The literature suggests several indicators and methods for the evaluation of patient engagement in the design of clinical trials. For example, Guarino et al measured participants’ understanding of the study consent form, using the Informed Consent Questionnaire‐4 questionnaire. The reading levels of the consent forms were assessed using Flesch‐Kincaid reading level scores.^70^ Rating the impact of patient engagement on study volunteer attitudes about aspects of the participation process (eg ease of understanding the informed consent form; convenience of study visits and procedures) is also suggested.^59^ Other studies suggest collecting data on the number of studies that gain research ethics committee approval,^12^ the number of protocol amendments^59^ and the number and type of patient‐reported outcomes.^61^ Furthermore, several studies have assessed study quality benefits, for example recruitment rates, using different quantitative methods.^40^, ^69^, ^70^ Iliffe, McGrath and Mitchell^40^ compared recruitment levels before and after the involvement of the public. Guarino et al^70^ also conducted a comparison; they assessed the effect of two different consent documents on recruitment levels using one consent form developed by a consumer focus group compared with another developed by the study investigators. Ennis and Wykes conducted a quantitative analysis of successful recruitment by studies where patient engagement was undertaken. A change in patient engagement over time was assessed by correlating study entry order (studies were ordered by the date identified) with the level of patient engagement. Additionally, suggested indicators include recruitment and retention rates among hard‐to‐reach populations,^61^ the number of dropouts for reasons other than adverse reactions, the total number of changes made to clinical trial communications as a result of patient feedback,^59^ and the number of studies completed within a particular time frame.^3^, ^61^ Validated questionnaires such as the Client Satisfaction Questionnaire‐8 measure overall satisfaction of study participants.^70^ Qualitative methods are mostly suggested for gathering information about participants’ experiences of taking part in a clinical trial.^15^

#### 3.1.3 Benefits of patient engagement in regulatory processes and health technology assessment (HTA)

We identified five unique benefits of patient engagement in regulatory processes and HTA, including four possible indicators. The benefits can be categorized into three dimensions: instrumental benefits, study uptake benefits and developmental benefits. Instrumental benefits are related to improving the relevance of assessment to making better quality decisions, for example higher accuracy in measuring needs and preferences of patients and better quality of assessment and relevance of reports to the local context.^71^, ^72^ Study uptake benefits refer to the usefulness of assessments for decision makers and the uptake of evidence by decision makers, for example gaining regulatory approval.^2^, ^73^ Developmental benefits include, for example, increasing the public's understanding of HTA and openness of decision processes.^72^

Literature suggests a few methods to evaluate the benefits of patient engagement in regulatory processes and HTA. Quantitative methods are suggested to assess study uptake benefits such as the time to response/approval of regulators and a change in the proportion of drugs recommended for reimbursement.^3^, ^74^ Furthermore, quantitative methods could be used to assess the perceived impact. For example, the European Medicines Agency has used a survey to assess the perceived added value of patient input in scientific advice processes and feedback.^75^ Qualitative methods can also be used to explore measures of change or uptake of patients’ input. For example, Abelson et al^76^ assessed how patients’ input informed the HTA process through document analysis, interviews and observations. Dipankui et al^77^ used semi‐structured interviews and document analysis (eg HTA reports, minutes) to evaluate how patient engagement changed the HTA report and its recommendations.

### 3.2 Costs and challenges of patient engagement in research and development

Limited studies have published costs and challenges. Of those which have, most studies reported increased time and costs for researchers and research institutions due to the practical aspects of planning and managing patient engagement. For example, there are increased time and financial costs from building relationships with the relevant community, setting up user groups, organizing and providing training and education for users and researchers, and the additional time needed for users to read and comment on documentation.^20^ Only two studies suggest that patient engagement could potentially result in a more homogenous sample or biases in recruitment.^24^, ^67^ For example, Blackburn et al^24^ reported that a more homogenous study sample may have been recruited, since the young contributors encouraged their friends to participate in a study on reproductive health in young people. Furthermore, Brett et al found that studies indicated that patient engagement led to scientific and ethical conflict in protocol design. Also, patient engagement may lead to tokenistic engagement and can lead to power struggles between researchers and patient partners.^20^ Furthermore, stakeholders have raised concerns that engaged patients may want to see their clinical trials succeed, and as a result, these patients may bias the study findings.^59^ It was also reported that a number of clinical research professionals fear that patient centricity is pushing them to discard traditional practices, including the use of blinded, randomized controlled clinical trials.^59^

Methods to assess costs include qualitative methods to gather insights into the perceived effort of engagement as well as a quantitative method to gather insights into financial costs. For example, the costs and consequences framework developed by Blackburn et al includes questions about costs for researchers such as total costs associated with recruiting patients involved, the total costs associated with training patients involved, the total costs associated with supporting patients, financial payment/rewards, total costs of expenses reimbursed to all patients for their involvement and other costs (including parking permits, room booking, audio‐visual, equipment). A separate questionnaire developed for patients includes questions about the hours spent on engagement, the costs they incurred (eg travel, child care, food and drinks, accommodation) and any costs related to arrangement and planning (for instance changed shifts at work or arranged care for a relative).^24^ Log sheets are also used to gather insights into time and costs.^27^ Open questions are used to gather insights into (non‐financial) negative impacts.^24^

### 3.3 Benefits, costs and challenges for stakeholders

Studies that assessed patient engagement for individuals and organizations mostly highlighted benefits, costs and challenges for patients engaged, with comparatively less published on the benefits and costs for other groups. Based on our review, suggested dimensions to measure the benefits, costs and challenges for the individuals and organizations involved relate to personal development, skills and knowledge, emotions and meaningful relationships, financial, performance and strategic value, transparency and awareness, trust and mutual respect. A summary of reported benefits and costs for stakeholders can be found in Table 6. Please refer to Appendices S1 and S2 for more detailed information on benefits, costs and challenges for patients and other stakeholders.

**Table 6:** Summary of benefits, costs and challenges per stakeholder group

Individuals and organizations	Benefits	Costs and challenges
Patient partners	Empowerment^8^, ^19^, ^20^, ^29^, ^30^, ^31^, ^55^, ^85^, ^86^ Enhanced well‐being^29^, ^30^, ^87^ Learning about research and gaining research and transferable skills^20^, ^24^, ^29^, ^55^, ^97^ Learning about own condition and treatment options^54^, ^79^ Enjoyment and satisfaction^22^, ^29^, ^55^, ^87^ Supportive, meaningful relationships^29^, ^31^, ^79^ Possible remuneration^8^, ^54^ Future prospects^29^, ^30^, ^79^, ^87^	Confusion due to lack of clarity about roles and procedures^67^, ^79^ Disappointment and frustration due to mismatched expectations^20^, ^27^ Stress due to lack of knowledge and confidence and a burden of responsibility^24^, ^79^ Overburdened^5^, ^29^, ^55^, ^79^ Investment of time and possibly own resources^4^, ^24^, ^67^, ^79^, ^88^ Possible reduction of welfare payments^24^
Society	Hope and trust in research/ers^29^, ^67^, ^79^ Funding and prioritization of research relevant to the community^3^ Potentially more, relevant drugs recommended for reimbursement^74^ Increased awareness of and advocacy for condition and associated research^67^, ^79^	Uncover or create conflict and power struggles in the community^79^ More time and resources^67^, ^79^, ^85^ Difficulty representing vulnerable/hard‐to‐reach groups^67^, ^79^
Research participants	Accessible information on all aspects of disease and treatment^24^, ^55^ More positive experience of research participation^2^, ^55^, ^80^	
Researchers	Learning about patients’ view of condition and patient engagement's effects on research^5^, ^8^, ^24^, ^26^, ^28^, ^29^, ^30^ Enhanced knowledge and skills^8^, ^28^, ^55^, ^79^ Fresh perspective on what research can achieve^19^, ^20^, ^22^, ^85^ Enjoyment and satisfaction^29^ Career benefits^29^, ^67^	Methodological concerns and costs^20^, ^32^, ^59^ Stress due to new ways of working with patients and advocacy groups and associated power struggles^4^, ^20^, ^32^, ^55^, ^67^, ^79^ More resource‐intensive research process^19^, ^55^, ^67^, ^79^, ^86^, ^87^, ^89^, ^90^
Research institutes	Increased research impact^24^ Enhanced reputation^24^	Diversion of research funds to patient engagement (opportunity cost in terms of funded researcher time, etc)^24^ IT and other support infrastructures 24
Research funders	More relevant funding decisions^24^ Increased transparency and accountability^55^, ^67^	Possible challenge to balance scientific integrity and relevant research^55^
Industry	More cost‐effective R&D^2^, ^85^, ^91^, ^92^, ^93^ More regulatory success^2^ Enhanced reputation^2^, ^31^ Better patient concordance with treatment^93^, ^94^ Enhanced knowledge^94^	More resource‐intensive R&D^20^
Regulators and health technology assessment bodies	Better understanding of real‐life context of products^71^ More efficient, relevant regulatory decisions^95^ Increased transparency and accountability^73^, ^74^ Mutual respect between regulators and consumers^73^	Increased uncertainty in policy‐making due to varied views^67^
Others (decision makers and health‐care providers)	More useful evidence for clinical and health policy decision making^30^	Uncertainty about how to take the study recommendations forward due to complexities of conflicting clinical and health system goals between clinicians, researchers, and users^55^

Multiple tools have been developed to assess the benefits and costs for stakeholders. The Evaluation Toolkit is a resource designed for practitioners of the health sector, produced after the completion of a rigorous systematic review of patient and public engagement evaluation tools.^78^ Boivin et al reviewed the tools and concluded that most tools were designed to collect information from patients and the public; very few instruments measure the perspectives of other stakeholder groups. The authors of the review reported that the outcomes of patient engagement were least often evaluated (55.6% of the tools), in contrast to the engagement process and context. The most common focus of tools that measure outcomes was on perceived, self‐reported impacts. Methods are qualitative (eg interviews, focus groups) and quantitative for perceived self‐reported benefits (eg surveys using Likert scales). Self‐administered questionnaires and surveys were the most common types of tools identified.^21^

## 4 DISCUSSION

To address the need for means of determining the “return on engagement,” the aim of this paper was to review the literature on monitoring and evaluation of patient engagement. This review identified a range of benefits, costs and challenges that patient engagement can have on R&D and describes several indicators associated with their monitoring and evaluation. In addition, we summarized the overall reported benefits, costs and challenges for stakeholders involved in patient engagement initiatives. In this section, we reflect on the indicators and methods found in this review and consider the review's methodological strengths and limitations.

### 4.1 Reflection on our findings

A total of 18 benefits and five costs of patient engagement at the three decision‐making points were identified in this review. These were grouped into 11 domains and mapped with 28 possible indicators for their evaluation. Little is known about the validity and performance of these indicators as most were suggested rather than applied, or used in single studies. Those studies mostly considered a single indicator (eg recruitment rate) for trying to answer a single question (eg Does patient engagement in research lead to better recruitment?). Measuring this may be feasible but may not be useful in predicting impact for other studies, as the factors influencing impact may differ. This has been noted by other authors.^36^ We argue that currently available indicators are of some use in measuring benefits, but are not sufficient to understand the pathway to impact, or whether the interaction between researchers and patients involved could lead to change in the external environment (eg research culture, structure and practice). We argue that the impacts of patient engagement can best be determined not by applying a single indicator, but a coherent set of measures. Given the importance of context and the complexity of evaluating patient engagement that this review illustrates, we are developing a monitoring and evaluation framework that considers various indicators for patient engagement practices in medicines research and development. This framework is informed by other frameworks and being tested in practice. We will publish our findings of working with a more coherent evaluation approach in medicines research and development shortly.

This review also looked at methods for evaluation. We identified several quantitative methods to measure the benefits of patient engagement; these mostly assess the benefits on study quality, study uptake and self‐reported benefits. Qualitative methods are mostly suggested for gathering information about experiences, attitudes and perceptions. We agree with others that there is a need for new evaluation methods and tools that focus on observable impact on the research process and benefits for those involved.^4^, ^21^, ^79^, ^80^ Some argue for broadly applicable, quantitative methods whilst others contend that more subjective, qualitative methods are necessary to capture the nuances of outcomes and impacts of patient engagement.^13^, ^17^, ^59^, ^76^ Universally applicable evaluation criteria that capture all aspects of engagement are supported for reasons of consistency, reliability and comparison across different projects.^16^ To build an evidence base, conceptual and practical guidance and some level of consensus on measurable impacts are needed. This has also been suggested by other authors.^13^, ^30^ However, whilst a standardized approach may be appealing to health research and development communities, it is problematic in the complex and contextually dependent arenas of patient engagement.^81^ It might inhibit capacity‐building in projects and makes changes difficult; arguably, this undermines the original rationale for patient engagement. The tension between obtaining comparable data on patient engagement by using metrics (standardized or agreed techniques for gathering information) and tailored participatory evaluative approaches should not be overlooked. By implication, it should be recognized that measures can be valued and applied differently in different contexts; therefore, we recommend discussing relevant and feasible indicators and methods per setting.

### 4.2 Strengths and limitations of this review

To our knowledge, this is the first literature review that attempts to capture the existing publications about the evaluation of patient engagement practice as it relates to medicines development. It both maps outcomes and impacts of patient engagement with suggested measures for each decision‐making point in R&D.

Very few publications refer to costs or negative impact of engagement, compared with positive findings. This may be because people tend not to report negative outcomes and impacts despite their being just as important. There were very few studies that considered patient engagement in the HTA process, and only, three publications were authored by (and for) the pharmaceutical industry. Furthermore, of the papers included in our review, very few reported that they had involved patients; therefore, the conclusions derived from the studies may be based on the perspectives of researchers. For this review, a meeting was held to discuss preliminary findings with a broad range of stakeholders in our project and the co‐authors of this paper work for patient representative groups and industry. We therefore feel that our findings may be considered relevant to a broader audience than a predominantly academic one.

Our focus on the measurement of impact of patient engagement in the development of medicines has resulted in several limitations to our review. Because this is a scoping review rather than a systematic review, we may have missed relevant articles. Our search focused on titles and abstracts of publications and three decision‐making points, which means that some articles (eg related to other time points) have been excluded. We specifically searched for outcomes and impact of patient engagement in the R&D of medicines; therefore, our paper does not include context or process indicators, or the indicators per stakeholder group. Furthermore, we cannot draw hard conclusions about the relationship between input, outcomes and impact with respect to the benefits and costs for the people and organizations involved in patient engagement. Finally, we had to exclude articles not published in English. Whilst we are aware that most publications on this topic are written in English originating from the UK and North America, we acknowledge that we may have missed relevant publications in other languages.

## 5 CONCLUSIONS

For patient engagement in the development of medicines to become standard practice at the key decision‐making points of priority setting, clinical trial design and regulatory and HTA processes, benefits need to be demonstrable to all stakeholders. This literature review has mapped benefits, costs and challenges with indicators in current literature. Discrete tools and methods for evaluation are less apparent, as is evidence of their application. The approaches to evaluation we found are largely qualitative, and our review suggests that there are few quantitative tools and no standardized approaches to assessing the outcomes and impact. The reported costs, challenges and benefits are largely congruent, with agreement that there is a need for consensus‐based monitoring and evaluation frameworks that include metrics.

We suggest that the development of a coherent set of measures warrants further investigation and that the benefits, costs and challenges of patient engagement for all stakeholders should be given more consideration (rather than the current focus on benefits for research). To this end, we will co‐develop and test an evaluation framework with stakeholders using a reflexive monitoring approach in real‐life cases of patient engagement in medicines research and development.

## CONFLICT OF INTEREST

None declared.

## Data Availability

The data that support the findings of this study are available from the corresponding author upon reasonable request.
