# Supplementary material for: Evaluating the “return on patient engagement initiatives” in medicines research and development: A literature review
Source: Health Expect. 2019 Sep 6;23(1):5–18. doi: 10.1111/hex.12951 (PMC6978865; doi:10.1111/hex.12951)
Supplement: Supplementary file 1 [file HEX-23-5-s001.pdf]

Annexe 1: Benefits, costs and challenges for patient partners

Benefits for patient partners (theme)	Suggested/assessed benefits for patient partners	Costs and challenges for patient partners (theme)	Suggested/assessed costs and challenges for patient partners
**Personal benefits**	Feeling listened to and valued[19,20,26,55,87,89,91,99]	**Personal costs**	Feeling limited influence/powerless, fear of tokenism/false appearance of inclusiveness[4,5,20,26,29]
	Increased self-confidence[3,5,19,20,22,28,29,55,85,87]		Mismatch of expectations, disappointment not given support in how to manage their care, lack of motivation to be involved in future research projects due to the failure of researchers to provide feedback, not able to learn how useful their input had been[20,27]
	Increased feeling of self-worth, empowerment[8,19,20,29–31,55,85,99]		Frustrations (e.g. with formal procedures/requirements, length of the project, frustrations due to assumptions that patients have a lack of knowledge and therefore not taken seriously, resistance to patient engagement/limited believes of some of the 'experts')[26,55,79]
	Increased hope and trust in research and researchers[3,19,20,31,54]		Confusion and conflict due to lack of clarity about their roles in the research, unease at the changing roles between users and health professionals[58,79]
	Increased well-being and mental health[29,30,87]		Feeling inexperienced, unable to contribute, lack of understanding research[29,79]
			Feeling left out of communications, feeling that more weight was put on issues expressed by those who were able to present their views more cogently than others[58,79]
			Opportunity to get personally exposed through inappropriate media coverage of the research project[29]
**Skills & knowledge**	Gaining/enhancing research skills (such as interviewing, questionnaire design, data-analysis)[5,29,55,79]	**Skills & knowledge**	Training required investment of time, resources and personal relationships[85]
	Gaining/enhancing transferable skills (such as listening, expressing themselves, collaboration, public speaking, computer skills, ability to channel anger into something constructive, ability to problem solve)[19,22,24,29,55,79]		
	Increased access to relevant research, understanding of research/clinical trial, research literacy[3,4,8,24,26,29,31,79,87,100]		
	Increased understanding of own condition, awareness of treatment options and how to access services[24,54,55,79]		
**Emotional and social benefits**	Enjoyment (e.g. working with group members, interaction)[22,26,29,55,87]	**Emotional costs**	Stress (e.g. burden of responsibility)[24,79]

	Satisfaction (e.g. ability to make a difference, sense of achievement, sense of ownership)[3,22,28,30,55,56]		Painful memories, upset by loss of their follow team members through a re-occurrence of illness[5,29,79]
	Meaningful activity (e.g. ability to give something back and doing something meaningful, being able to concentrate on something else then themselves)[24,29,31,79]		Difficulties to cope with insensitive views and opinions expressed by professionals[29]
	Enhanced network and friends (e.g. meeting new people, enhanced network, making new friends, sense of community)[24,29,31]		Feeling over burned[79,5,29,55]
	Support (e.g. sense of mutual support by fellow users)[29,79]		Feeling anxious about how much research costs[29]
**Financial and other benefits**	Paid job, regular employment[8,29]	**Financial and other costs**	Financial burden (e.g. paid work, child care, informal care, travel costs if not reimbursed)[24,58]
	Future opportunities to participate in research[30]		Possible effects on welfare payments[24]
	May improve chances of future employment and enrollment in education[29,30,79,87]		Time consuming. Difficulties fitting with work and around other life commitments[4,79,89]
**Real-world benefits**	More drugs recommended for reimbursement[74]		
	Access to funding for bringing researchable topics to the research agenda that otherwise may not be taken into consideration[3]		
	Research and research outcomes address patients' genuine unmet needs, development of health care and therapies that are more representative of patient's needs[3,91]		

Annexe 2: Benefits, costs and challenges for the stakeholder groups

Stakeholder	Suggested/assessed benefits	Suggested/assessed costs and challenges
**Researchers**	• Motivational benefits (e.g. research seem worthwhile, improved confidence and trust, enthusiasm, inspiration to work toward solutions)[8,22,24,26,28,31,79]<br>• Performance benefits (e.g. increased enrolment in studies and decreased attrition; improved data collection tools; improved dissemination of study findings and mobilisation of findings)[19]<br>• Developmental benefits (e.g. greater understanding and insight into research area; better alignment of research objectives through priority-setting activities)[19,79,85]<br>• Acquired/enhanced knowledge (e.g. understanding of the condition of interest, ideas for new research, how to encourage participation, how their project design is potentially off-putting to participants)[5,8,24,26,28–30]<br>• Acquired/enhanced skills (e.g. increased their skills in communicating with a lay audience, facilitation skills)[8,28,55,79]<br>• Changed preferences/priorities (e.g. misassumptions about what topics are most relevant and important to patients, reality check, changed ideas about outcome measures)[5,28,53,79]<br>• Changed attitude to involvement, sense of a more equalised relationship sense of social justice[8,28–30,55,79]<br>• Enjoyment and satisfaction (e.g. rewarding process, made new friends, fun)[29]<br>• Career benefits (e.g. because they received public recognition for their community work, internal recognition and validation from their employers)[29,58]	• Financial costs (e.g. more resources and time, slowing the pace of research)[19,26,55,58,79,87,91,99]<br>• Emotional costs (e.g. increased pressure/stress, sensitivity to criticism, requires new skills, changes in working practice, tension between what constitutes a good research study)[24,29,55,58,79]<br>• Fear of tokenism/false appearance of inclusiveness, power struggles between researchers and users[4,20,32,55,58,79]<br>• Methodological costs (e.g. patients may bias the study findings, irrelevant community concerns and issues, which would make the research unfeasible, concerns that users may come with their own lobbying agenda)[20,32,59]<br>• Concerns about what contribution users could make to a research project and concern over the competence of users to assist with research[79]
**Research institution**	• Increased impact of research[24]<br>• Recognition as a centre with expertise and experience of involving patients and public in research (raising the institution's profile)[24]	• Costs (e.g. diversion of research funds to patient engagement, opportunity cost in terms of funded researcher time, etc.)[24]

		• IT and other support infrastructures/resources (including printing & internal room bookings)[24]
**Research funder**	• Avoiding devoting resources to a topic which is not important (e.g. exploring an intervention which is not appealing to service users)[24]<br>• Increased transparency and accountability of funding organisations[55,58]	• Challenge that funders and ethical committees look for scientific integrity, whereas user-led research focus on making research „real"[55]
**Industry**	• Decreased costs (e.g. by avoidance of amendments, increased patient trial experience, reduced time to complete the study, greater market share through more advanced technologies)[2,85,93–95]<br>• Methodological benefits (e.g. more efficient trial design, more feasible and tolerable clinical trial designs, increased quality of data/statistical power, improved recruitment and retention)[2,96]<br>• Increase probability of regulatory success (e.g. by better demonstrations of efficacy and safety)[2]<br>• Strategic value (e.g. reputational boost, mutual respect, patient satisfaction)[2,31]<br>• Acquired/enhanced knowledge (e.g. knowledge of conditions, interventions, better understanding of how patients view the benefits of treatment and the harms and risks they pose)[31,96]<br>• Better adherence to medication regimens. Patient preference for and adherence to developed products or services may be greater[95,96]	• Negative impact on clinical trial budgets, duration, and efficiency. Return on investment (ROI) expectations need to take a reasonably long-term view[59]
**Regulators and health technology assessment bodies**	• Increased transparency in public decision-making and trust in regulatory processes[73,74]<br>• Mutual respect between regulators and the community of patients and consumers[73]<br>• Enriches the content of reports, recommendations, quality of the opinion given by the scientific committees[73,77]<br>• Being able to provide clear and useful information[73]<br>• Development of medicines more efficiently, ensuring that effective, safe medicines reach those who need them as quickly as possible[97]<br>• Better understanding of technologies' impact in real life context and also the quality of life aspects', leading to decisions that meet patients' needs[71]	• May include increasing the uncertainty of policy making when different viewpoints call for different policy responses[58]

**Society**	• Mutual respect between researchers and the community, greater intercultural understanding by all parties[29,79]<br>• Increasing the acceptability and trust of the research in the community, this may give research credibility in the community[29,58,79]<br>• Resolving conflict between researchers and the community[29,79]<br>• Increase the awareness of the disease or condition in the community, greater knowledge of and better distribution of information on diagnosis and treatment in the community, potentially leading to a better informed patient population[58,79]<br>• Users became advocates of the research in the community, sense of community ownership of research, willing to act on findings[29,58,79]<br>• Can gauge opinion from broad(er) sections of the community and introduce a broader range of views when committees consult peers[85]	• Conflict and power struggles within the community[79]<br>• May increase the time and cost burdens of the community organisations involved[58,79,85]<br>• May be difficulty representing those in the community who are severely disabled or severely ill, because of their health status[58,79]
**Research participants**	• Improved patient experience during clinical trials/research process[2,29,55,80]<br>• The research process might be seen to be more acceptable, user-friendly and sensitive, less burdensome[55,58,94]<br>• Participants may be more willing to talk and raise issues and be more willing share their true experiences and views, emotional support by peer researchers[29,55,58]	• Sharing issues with peer interviewers can be seen as inappropriate and could lead to tensions[55,58]
**Others (decision-makers and healthcare providers)**	• More useful evidence for clinical and health policy decision-making[30]<br>• Helps legitimize research findings used to change policy[55]	• Complexities of conflicting clinical and health system goals between clinicians, researchers, and users, and the constant changes of health and research processes and systems leading to uncertainty about how to take the study recommendations forward[55]

Annexe 3: Benefits of patient engagement in research priority-setting, including suggested indicators, methods and tools

Suggested and/or assessed outcomes and impact	Suggested and/or used* indicators	Methods and tools used in published studies to assess outcomes and impact of patient engagement
**Domain: usability benefits**<br><br>**Research topic, priorities become more appropriate, based on patients' needs[3,4,15,17,20,22,23,29,30,50–55]**<br><br>**Research questions, outcomes/end-points, interventions and medical technologies become more relevant and usable for patients[8,23,24,29,30,32,41,50,51,53,55,64,65,68,94]**	Quantitative:	
	*Rating of partner influence across study phases[23]	Survey, 1 item, 4 point Likert scale from "none" to "A great deal" (investigator-reported)[23]
	Ratings of relevance made by key stakeholder groups outside the research team including service users and grant funders[15]	
	Ratings of perceived relevance or importance of studies and whether a given study addresses an unmet medical need[59]	
	Successful priority-setting leads to increases in satisfaction over multiple decision cycles. Stakeholder acceptance is indicated by continued willingness to participate in the process (i.e. 'buy-in') as well as the degree of contentment with the process[101]	
	Qualitative:	
	Explore similarities and differences in research priorities. Use qualitative methods to explore perceptions of relevance[15]<br><br>Proposed impact assessment question: does involving service users on research priority development lead to proposals that are perceived to be more relevant to key stakeholder groups?[15]	Patients with diabetes were invited to focus groups to identify research priorities; results were analysed using the constant comparative method. Results were compared with current expert-led research priorities in diabetes[63]
	*Assess the influence of public involvement in the research agenda setting process[103]	Triangulated approach, involving documentary data analysis, video and cassette tape analysis, (direct) observation, and semi-structured interviews. Document analysis included grant applications, reports, minutes to compare patient input and responsiveness to ideas[103]
	*Types of gaps documented as important to patients and other stakeholders that were not previously identified[61]	Database review and document review[61]
	*How many submitted topics score well on the topic selection patient-centeredness criterion[61]	Database review and document review[61]

	Mixed methods	
	*Perspectives incorporated into topic selection process. [61]<br>Stakeholder perceptions of topic generation and research prioritization process, such as, perceived influence on the content of the topic database<br>Relative contributions of the patients and stakeholders in ranking submitted topics<br>Panel members' perceptions of the topic generation and research prioritization process<br>Indicators of dynamics in the panel discussion<br>Number and type of stakeholders submitting topics to PCORI	Focus groups, surveys, and database review[61]
**Domain: societal benefits**<br><br>**Resource allocation become more appropriate, based on patients' needs[30]**<br><br>**Influence on funding decisions via patient engagement in peer review[29]**<br><br>**Shifted priorities and/or reallocated resources[101]**	Quantitative:	
	*Comparison of academic and lay scores assigned to research proposals[62]	The scores assigned to research proposals were on a 5-point scale to one decimal point, with 1.0–1.5 being Outstanding, 1.5–2.0 being Excellent, 2.0–2.5 being Very Good, 2.5–3.5 being Good, and 3.5–5.0 being Acceptable. Pearson product-moment correlation coefficient was used to assess the degree of relationship between mean consumer and mean scientist scores for the population of proposals[62]
	*Perceptions of public influence on the review panel[62]	Before (pre-panel) and after (post-panel) opinion questionnaires, consisting of open and closed response questions. The pre and post-panel questionnaires were matched by the participants' self-assigned code. Chi-square tests were used to analyse responses[62]
	Qualitative:	
	None reported	
**Domain: funding benefits**<br><br>**Motivation to seek for funding, new funding and funding opportunities, research more fundable, enhanced credibility[25,29–32,56–58]**	Quantitative:	
	Number of studies that had success in gaining research funding[12]	
	Qualitative:	
	None reported	

Annexe 4: Benefits of patient engagement in the design of clinical trials, including suggested indicators, methods and tools

Suggested and/or assessed outcomes and impact	Suggested and/or used* indicators	Methods and tools used in published studies to assess the outcomes and impact of patient engagement
**Domain: study quality benefits**<br><br>**Recruitment rates and retention improves, diversity of research subjects increased, adherence to the protocol[2–4,8,15,17,20,23,24,26,27,29–32,40,41,52,55,56,66,69,92–94]**<br><br>**Faster study completion as a result of improved recruitment and adherence to protocol[2,23]**<br><br>**Decrease costs as a result of improved recruitment, retention and trial experience[2,3,93]**	Quantitative:	
	*Recruitment rates[2,3,12,40,59]	Quantitative comparison of recruitment levels before and after the involvement of the public[40]<br><br>Quantitative comparison of the effect of two different consent documents (one developed by a consumer focus group of Gulf War veterans versus one developed by the study investigators) on recruitment levels. The associations between type of consent document and recruitment and adherence in the parent trial were analyzed by chi-square and t-statistic[70]<br><br>Quantitative analysis of study success and patient involvement over time. Assessed by correlating study entry order with level of patient involvement using Pearson's product moment. Predictors of levels of patient involvement were explored using multinomial logistic regression. Predictors of successful recruitment were explored using binary logistic regression again with a backward conditional method to identify variables that predicted whether a study hit the recruitment target (90%). The independent variables were funder, clinical study group, complexity, randomisation (yes/no), follow-up (yes/no) and study type (observational/interventional/both)[69]
	Increased levels and diversity of service user involvement[15]<br><br>Proposed impact assessment question: does patient involvement through Advisory Panel members leading the development of The Spectrum Centre recruitment pathways lead to an increase in the number and diversity of service users recruited to take part in The Spectrum Centre activities?[15]	

	Suggested performance measures that can be used to largely compare studies that do and do not include patient-centric initiatives.[59]<br>Screen failure rates<br>Number of procedures per visit<br>Number of protocol amendments<br>Number of missed visits<br>Number of study volunteers who drop out prematurely for reasons other than adverse/serious adverse reactions<br>Number of study volunteers completing participation as a percentage of those who enrolled	
	*Changes to study design resulting from engagement:[61]<br>number and type of patient reported outcomes (PROS)<br>changes to outcomes resulting from engagement<br>recruitment and retention rates<br>recruitment and retention rates among hard to reach populations,<br>study participants' experiences in the research	Self-report data from PCORI funded researchers and their patient and stakeholder partners: intern process reports and WE-ENACT PCORI survey[61]
	Number of studies completed within a particular time frame/time saved in trial timelines[3]	
	* Expected Net Present Value(ENPV)[2]	Impact of patient engagement on Expected Net Present Value for a typical oncology development program entering phase 2 or phase 3 assessed based on avoidance of amendment, improving patient experience and expected probability of technical and regulatory success[2]
	Qualitative:	
	None reported	
**Domain: ethical benefits**<br><br>**Design/process more appropriate, inclusive, sensitive and ethical[8,17,29,30,52,55,58]**	Quantitative:	
	Number of studies that had success in gaining research ethics approval[12]	
	*Ratings of overall satisfaction among participants in a particular study[59,70]	Client Satisfaction Questionnaire-8[70]

	Comparison of before-and-after ratings of volunteer satisfaction between multiple studies that did and did not implement patient-centric initiatives[59]	
	Ratings (e.g. ‘‘very,’’ ‘‘somewhat,’’ ‘‘not very,’’ ‘‘not at all’’) of the impact that specific patient-centric initiatives had on study volunteer attitudes about aspects of the trial (e.g. ease of understanding the informed consent form; convenience of study visits and procedures) compared with studies that have not implemented patient engagement initiatives[59]	
	Qualitative:	
	Feeling of satisfaction among study participants[15]	Suggested: Interviews with study participants to understand their experiences of taking part[15]
**Domain: methodological benefits**<br><br>**Appropriate wording and timing of research instruments such as questionnaires and interventions[4,17,20,22,24,25,27,29,31,55,56,58,64–66,68]**<br><br>**Improved information accessibility, lay summaries, information sheets, consent form, recruitment materials[4,20,24,25,29,31,40,55,56,58,68]**	Quantitative:	
	Total number of changes that have been made to clinical trial communications as a result of patient/study volunteer feedback[59]	
	Reading level of research documents/instruments[34,70]	The reading levels of the consent forms were assessed using the Flesch-Kincaid reading level scores[70]
	*Measuring self-reported participant understanding[70]	Informed Consent Questionnaire-4 (ICQ-4), a validated four-item scale scored from 0 to 1[70]
	Qualitative:	
	None reported	

Annexe 5: Benefits of patient engagement in regulatory processes and health technology assessment, including suggested indicators, methods and tools

Suggested and/or assessed outcomes and impact	Suggested and/or used* indicators	Methods and tools used in published studies to assess the outcomes and impact of patient engagement
**Domain: instrumental benefits**<br><br>**Instrumental benefits such as higher accuracy in measuring needs and preferences of patients and higher reliability, better quality of assessment and comprehensive information, relevance of reports to the local context[71,72]**	Quantitative:	
	*Perceived impact of patient involvement in health technology assessment[71]	Survey - health technology assessment bodies and patient organizations[71]
	Qualitative:	
	*Members perceptions about how their input was used; changes to documents; member reflections on how patient input informed the process[76]	Document analysis, telephone interviews with panel members, observations[76]
	*Evaluation of changes in HTA reports and its recommendations due to patient involvement (consultation and direct participation) in the assessment of alternative measures[77]	Semi-structured interviews with stakeholders: caregivers, healthcare managers, patient representatives, health technology assessment unit members, researchers, and members of the local scientific committee. Content analysis of two reports and other documents that were produced[77]
**Domain: study uptake benefits**<br>**Gaining regulatory approval/availability of new drugs and technologies[2,73]**	Quantitative:	
	Time to approval/response of the regulators[3]	
	Change in the proportion of drugs recommended for reimbursement[74]	
	Qualitative:	
	None reported	
**Domain: developmental benefits**<br><br>**Increased knowledge and public awareness of products[72]**<br>**Democratic accountability and transparency[72]**	Quantitative:	
	None reported	
	Qualitative:	
	None reported	
